# Dual Role for the *O*-Acetyltransferase OatA in Peptidoglycan Modification and Control of Cell Septation in *Lactobacillus plantarum*

**DOI:** 10.1371/journal.pone.0047893

**Published:** 2012-10-26

**Authors:** Elvis Bernard, Thomas Rolain, Blandine David, Guillaume André, Vincent Dupres, Yves F. Dufrêne, Bernard Hallet, Marie-Pierre Chapot-Chartier, Pascal Hols

**Affiliations:** 1 Institut National de la Recherche Agronomique, UMR1319 Micalis, Jouy-en-Josas, France; 2 AgroParisTech, UMR Micalis, Jouy-en-Josas, France; 3 Biochimie et Génétique Moléculaire Bactérienne, Institut des Sciences de la Vie, Université catholique de Louvain, Louvain-la-Neuve, Belgium; 4 Bio and Soft Matter, Institute of Condensed Matter and Nanosciences, Université catholique de Louvain, Louvain-la-Neuve, Belgium; University of Kansas Medical Center, United States of America

## Abstract

Until now, peptidoglycan O-acetyl transferases (Oat) were only described for their peptidoglycan O-acetylating activity and for their implication in the control of peptidoglycan hydrolases. In this study, we show that a *Lactobacillus plantarum* mutant lacking OatA is unable to uncouple cell elongation and septation. Wild-type cells showed an elongation arrest during septation while *oatA* mutant cells continued to elongate at a constant rate without any observable pause during the cell division process. Remarkably, this defect does not result from a default in peptidoglycan *O*-acetylation, since it can be rescued by wild-type OatA as well as by a catalytic mutant or a truncated variant containing only the transmembrane domain of the protein. Consistent with a potential involvement in division, OatA preferentially localizes at mid-cell before membrane invagination and remains at this position until the end of septation. Overexpression of *oatA* or its inactive variants induces septation-specific aberrations, including asymmetrical and dual septum formation. Overproduction of the division inhibitors, MinC or MinD, leads to cell filamentation in the wild type while curved and branched cells are observed in the *oatA* mutant, suggesting that the Min system acts differently on the division process in the absence of OatA. Altogether, the results suggest that OatA plays a key role in the spatio-temporal control of septation, irrespective of its catalytic activity.

## Introduction

Peptidoglycan (PG), the major cell-wall compound in Gram-positive bacteria, is composed of a polymer of the disaccharide *N*-acetylmuramic acid (MurNAc)-(β-1,4)-*N*-acetylglucosamine (GlcNAc) associated with peptidic stems linked to MurNAc [1]. The glycan strands are the target for a range of modifications. In *L. plantarum*, the addition of an *O*-linked acetyl group on both MurNAc and GlcNAc, as well as the presence of a covalently-linked glycopolymer on MurNAc called wall teichoic acid (WTA), have been reported [2], [3]. MurNAc *O*-acetylation and WTA anchoring take place on the same C6-OH of MurNAc and are mutually exclusive [4], [5].

In Gram-positive bacteria, MurNAc *O*-acetylation is catalyzed by the membrane-bound OatA protein, an *O*-acetyltransferase composed of 11 transmembrane segments at the N-terminus and a surface-exposed *O*-acetyltransferase catalytic domain at the C-terminus [3], [6]. The N-terminal transmembrane domain of the protein is thought to transport the acetyl donor, possibly acetyl-CoA, through the membrane [6]. In most species, MurNAc *O*-acetylation is described as to increase resistance to lysozyme, β-lactams, and endogenous autolysins [3], [6]–[9]. The level of this PG modification was shown to increase under specific conditions such as the viable but non-cultivable (VBNC) state of *Enterococcus faecalis* [10]. In addition, Veiga et al. [11] have shown that *oatA* overexpression in *Lactococcus lactis* results in growth arrest. The authors hypothesized that over-*O*-acetylated PG becomes too robust to sustain growth in this species. In *L. plantarum*, we have recently shown that MurNAc over-*O*-acetylation has different consequences. It has a modest effect on growth and activates autolysis through the LytH enzyme, a putative *N*-acetylmuramoyl-L-alanine amidase [3]. In addition, *L. plantarum* harbored *O*-acetylation on GlcNAc which is catalyzed by the OatB protein that displays a similar predicted topology than OatA [3]. We showed that OatB overproduction is toxic while its depletion increases the activity of the *N*-acetylglucosaminidase Acm2, which is the major autolysin of *L. plantarum* [3].

WTA is the second major polymer of the cell wall and consist in a backbone of poly(alditol-phosphate) that is decorated by different substitutions (D-alanylation and glycosylation) [12]. Depending on the nature of the backbone, synthesis of WTA is under the control of the *tag* genes (in the case of polyglycerolphosphate WTA) or the *tar* genes (in the case of polyribitolphosphate WTA) [4]. WTA were thought to be essential for growth [13]. However, abolition of their synthesis was successfully achieved in various species by the inactivation of *tagO*, the gene that is required for the first step of WTA synthesis [4], [14], [15]. WTA were reported as important morphogenesis actors [2], [15]. Indeed, they are involved in proper cell elongation, septum positioning, and localization of different PG maturing enzymes. In *S. aureus*, WTA were recently shown to recruit PBP4 at the septum and are consequently involved in the level of PG cross-linking [16]. In *Bacillus subtilis*, WTA are required to position the PG hydrolase LytF at the septum [17], thereby controlling its activity. In *L. plantarum*, WTA were shown by atomic force microscopy to localize in the cylindrical part of the cell wall and their absence was found to disturb both cell elongation and cell division events, suggesting a role in the control of cell morphometry and the recruitment of the cell division machinery in this species [2].

To our knowledge, the contribution of MurNAc *O*-acetylation and its dedicated *O*-acetyltransferase OatA to cell morphogenesis has never been investigated. Here, we show that the OatA protein is a key actor in the spatio-temporal control of septation in *L. plantarum*, independently of its O-acetyltransferase activity. The protein is predominantly localized at the septum in a complementary pattern of mature WTA. Depletion and overproduction of wild-type OatA or defective variants were found to specifically alter elongation-septation uncoupling, septum positioning, and activity of the MinCD division inhibitor.

## Materials and Methods

### Bacterial Strains, Plasmids, and Growth Conditions

The bacterial strains and plasmids used in the present study are listed in Table 1. Plasmids were constructed in strain MC1061 of *Escherichia coli*. *E. coli* was grown in LB medium [18] with shaking at 37°C. *L. plantarum* was grown in MRS broth (Difco Laboratories Inc., Detroit, MI) at 30°C. When required, chloramphenicol (10 µg/ml for *E. coli* and *L. plantarum*) and ampicillin (100 µg/ml for *E. coli*) were added to the media. Solid agar plates were prepared by adding 2% (w/v) agar to the medium. Nisin A (Sigma, Bornem, Belgium) was used at a concentration ranging from 2.5 to 20 ng/ml for different levels of induction of genes under the control of the *nisA* expression signals as previously described [3]. For cell morphology analysis, cells were routinely harvested from cultures in the exponential growth phase (OD_600_ between 0.6 and 0.8).

**Table 1:** Bacterial strains and plasmids.

Strain/plasmid	Characteristic(s)#	Reference/Source
**Strains**		
***Lactobacillus plantarum***		
**NZ7100**	WCFS1 *lp_0076::nisRK*	
**EB002**	NZ7100 *oatA::lox72*	[3]
**EB003**	NZ7100 *oatB::lox72*	[3]
***Escherichia coli***		
**MC1061**	F^−^ Δ(*ara-leu*)7697 [*araD139*]_B/r_ Δ(*codB-lacI*)3 *galK16 galE15* λ^−^ e14^−^ *mcrA0 relA1 rpsL150* (strR)*spoT1 mcrB1 hsdR2*(r^−^m^+^)	[32]
**Plasmids**		
**pNZ8048**	Cm^r^, shuttle vector containing P*_nisA_* promoter and start codon in NcoI site	[33]
**pGIEB003**	Cm^r^, pNZ8048 derivative containing *oatA* gene in transcriptional fusion	[3]
**pGIEB011**	Cm^r^, pNZ8048 derivative containing *oatA* ^D510A/S511A^ gene in transcriptional fusion	[3]
**pCS2Venus**	Amp^r^, pCS2 derivative containing the YFP (Venus) fluorescent protein	[34]
**pBID008**	Cm^r^, pNZ8048 derivative containing the YFP (Venus) fluorescent protein encoding gene	This study
**pGIEB018**	pBID008 derivative with OatA transmembrane domain fused to the N-terminus of YFP	This study
**pGIEB019**	pBID008 derivative with OatB transmembrane domain fused to the N-terminus of YFP	This study
**pGIEB020**	pBID008 derivative with MinC fused to the N-terminus of YFP	This study
**pGIEB021**	pBID008 derivative with MinD fused to the N-terminus of YFP	This study

# Cm^r^ and Amp^r^ indicate resistance to chloramphenicol and ampicillin, respectively.

### DNA Techniques and Electrotransformation

General molecular biology techniques were performed according to the instructions given by Sambrook et al. [18]. Electrotransformation of *E. coli* was performed as described by Dower et al. [19]. Electrocompetent *L. plantarum* cells were prepared as previously described [20]. PCR were performed with Phusion high-fidelity DNA polymerase (Finnzymes, Espoo, Finland) in a GeneAmp PCR system 2400 (Applied Biosystems, Foster City, CA). The primers used in this study were purchased from Eurogentec (Seraing, Belgium) and are listed in Table S1 in the supplemental material.

### Construction of YFP Fusions for Protein Localization and Overproduction

The *yfp* gene (Venus) from plasmid pCS2Venus was amplified by PCR using the primers NcoIVenus4X4-SacIVenus4X4 (Table S1). The PCR amplicon (808 bp) was restricted by *Nco*I and *Sac*I and then cloned into *Nco*I/*Sac*I-restricted pNZ8048 vector [21]. The resulting pGIBD008 plasmid contains the *yfp* gene under the control of nisin-inducible P*_nisA_* promoter [21]. DNA fragments coding for OatA and OatB transmembrane domains and their associated RBSs were amplified by PCR using primer pairs 5′oatAPstI/3′TMoatAXbaI and 5′oatBPstI/3′TMLoatBXbaI (Table S1), respectively. Both PCR amplicons were restricted by *Pst*I and *Xba*I and then cloned into the *Pst*I/*Xba*I- restricted pGIBD008 vector, leading to expression plasmids pGIEB018 and pGIEB019, respectively. The two generated constructs code for fusion proteins between the first 10 transmembrane segments of Oat proteins and YFP, which is located at the C-terminus (OatA^TM1–10^-YFP and OatB^TM1–10^-YFP, respectively). The *minC* and *minD* ORFs were amplified by PCR using primer pairs 5′minC_NcoI/3′minC_XbaI and 5′minD_NcoI/3′minD_XbaI, respectively (Table S1). The *Nco*I/*Xba*I restricted PCR fragments were cloned in the *Nco*I/*Xba*I restricted pGIBD008 vector, leading to expression plasmids pGIEB020 and pGIEB021, respectively. The two constructs encode YFP fusions with the fluorescent partner at the C-terminus. The four expression vectors were electrotransformed in different *L. plantarum* genetic backgrounds for complementation, overexpression, or localization studies.

### X-ray Photoelectron Spectroscopy

Cells were collected from exponentially growing cultures, resuspended in MilliQ water (Millipore) and directly lyophilized. XPS analyses were performed on a Kratos Axis Ultra spectrometer (Kratos Analytical) equipped with a monochromatized aluminium X-ray source. The angle between the normal to the sample surface and the electrostatic lens axis was 0°. The analyzed area was ∼700×300 µm. The constant pass energy of the hemispherical analyzer was set at 40 eV. The following sequence of spectra was recorded: survey spectrum, C_1s_, N_1s_, O_1s_, P_2p_, S_2p_ and C_1s_ again, to check the stability of charge compensation as a function of time and the absence of degradation of the sample during the analysis. To assess the level of surface contamination, sorbitol was included in the analysis, starting from the freeze-drying process. Binding energies were calculated with respect to the C-(C,H) component of the C1s peak of adventitious carbon fixed at 284.8 eV. Following subtraction of a linear baseline, molar fractions were calculated (CasaXPS program, Casa Software) using peak areas normalized on the basis of acquisition parameters, sensitivity factors and the transmission function provided by the manufacturer.

### Microscopy

Optical microscopy analysis was performed using a Reichert Jung Polyvar microscope and a Nikon digital sight DS-U1 camera for time-lapse experiments and Bright field microscopy. Fluorescent and phase-contrast images were acquired with an Axio observer Z1 inverted microscope (Carl Zeiss). FM4–64 (Molecular Probes, Leiden, The Netherlands) and DAPI (Sigma, Bornem, Belgium) stainings were performed as previously described [2]. Analyses of micrographies were performed using the Axiovisio 4.8. software (Carl Zeiss).

Atomic force microscopy (AFM) images were obtained in acetate buffer (150 mM in acetate, pH 4.75) at room temperature, using a Nanoscope V Multimode AFM with MSCT cantilevers (Veeco Metrology Group, Santa Barbara, CA). Cells were immobilized by mechanical trapping into porous polycarbonate membranes (Millipore, Billerica, MA) as previously reported [2].

## Results

### The *L. plantarum oatA* Mutant is Affected in the Control of Cell Length and Cell Cycle

**Figure 1:**
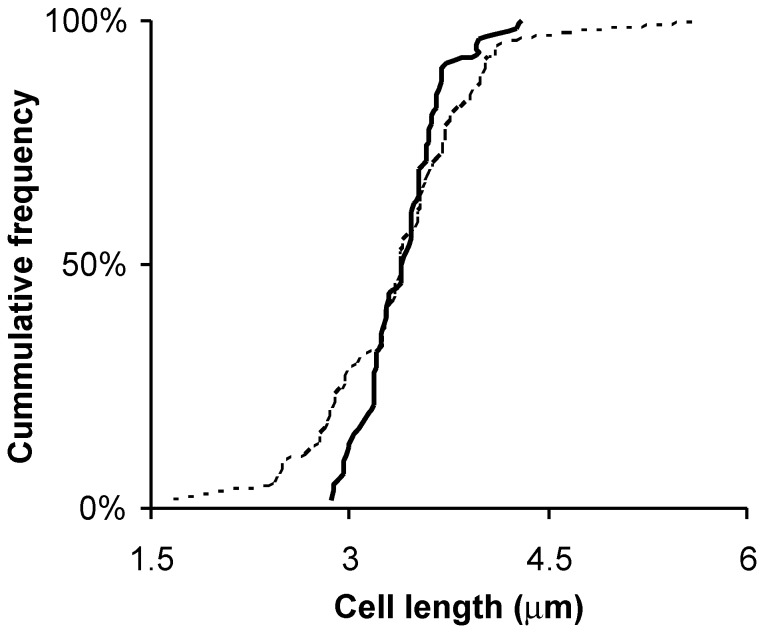
Cumulative frequency of the length (µm) of *L. plantarum* dividing cells. WT and *oatA* mutant strains (n = 56 and n = 73, respectively) are represented by solid and dashed lines, respectively. Only the sub-population of dividing cells is presented. Cells were observed at an OD_600_ of 2.0, corresponding to the end of the exponential growth phase.

In order to evaluate the contribution of MurNAc *O*-acetylation to cell shape maintenance in *L. plantarum*, the morphology of *oatA* mutant cells was examined by light microscopy. Mutant cells perfectly maintained their rod shape without any visible cell defect whatever the growth stage (data not shown). The average cell lengths of wild type and *oatA* mutant cells in the global population were quite similar (2.2 µm (n = 322) and 2.0 µm (n = 352), respectively), as well as their respective cell length distribution (data not shown). However, the sub-population of dividing cells with a visible cell invagination at mid-cell (∼ 20% of the global population at an OD_600_ of 2.0) displayed a much wider length distribution in the *oatA* mutant than in the wild-type strain (Figure 1). Dividing wild-type cells showed a length variation of about 1 µm (from 3 to 4 µm), whereas dividing *oatA* mutant cells had a length variation of 3 µm (from 2 to 5 µm) (Figure 1).

**Figure 2:**
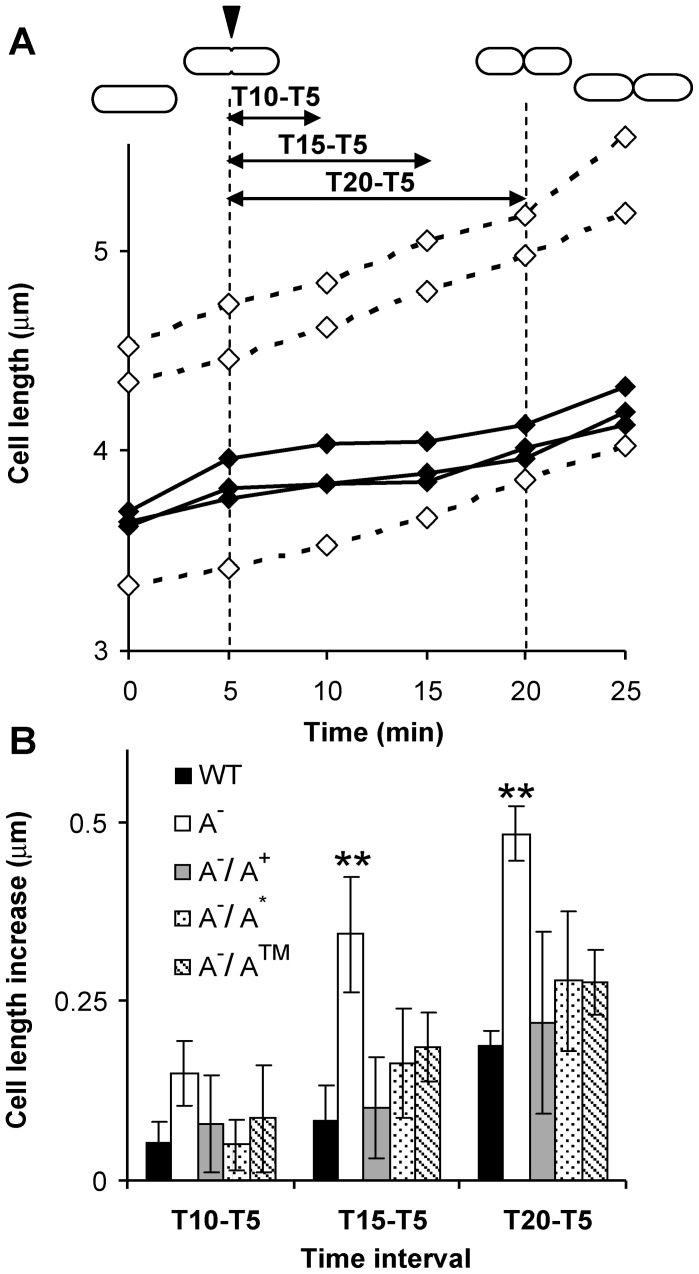
Study of the uncoupling between elongation and septation phases by time-lapse experiments. A) Cell length (µm) of the mother cell during the division process for the wild type (solid line) and the *oatA* mutant (dashed line). Each line corresponds to one individual cell. Time (min) was arbitrarily fixed to zero at the last view before any detectable cell invagination in bright field. Correspondences between time and division state are drawn upside of the graph for the wild type. One representative time-lapse experiment of three independent experiments for each strain (n ≥3 for each). All examined cells of the wild type and the *oatA* mutant (n = 15 for each) display their respective phenotype. B) Cell length increase (µm) measured during the division process after 5 min (T10–T5; T5, initial invagination), 10 min (T15–T5), and 15 min (T20–T5; T20, final septation). Time intervals are reported in panel A. Symbols: WT, wild-type; A^−^, *oatA* mutant; A^−/^A^+^ *oatA* mutant complemented with *oatA* ^WT^; A^−/^A^*^ *oatA* mutant complemented with *oatA* ^D510A/S511A^; A^−/^A™, *oatA* mutant complemented with *oatA* ^TM1–10^ *::yfp*. All the variants of *oatA* are expressed at low basal level in absence of the nisin inducer. Mean values of 5 cells in each time-lapse experiment. Significance based on *t*-test; **, p-value <0.01.

In order to closer examine the cell cycle of these two strains, time-lapse experiments were carried out (Figure S1). As can be seen in figure 2, wild-type cells showed an elongation arrest during the division process (Figure 2A) with a maximal length increase of ∼0.2 µm (Figure 2B, WT), which roughly corresponds to the septum thickness. In contrast, *oatA* mutant cells continued to elongate at a constant rate without any observable pause during the cell division process (Figure 2A) with a maximal length increase of ∼0.5 µm (Figure 2B, A ^−^). These observations showed that there is a temporal uncoupling between elongation and division in the wild type and that the *oatA* mutant is affected in this process.

### The Absence of the OatA Protein but Not *O*-acetylation is Responsible for the Altered Cell Cycle

With the aim to perform complementation experiments, the wild-type copy of the *oatA* gene (*oatA* ^WT^) and a mutated gene encoding an OatA-defective protein (OatA^D510A/S511A^) were expressed in the OatA-deficient strain [3]. The OatA^D510A/S511A^ protein has been mutated in two conserved residues of the catalytic domain and was shown to have completely lost its *O*-acetyltransferase activity [3]. These two genes were cloned under the control of the nisin-inducible P*_nisA_* promoter. In *L. plantarum,* this promoter confers a low level of basal expression in the absence of inducer [22]. Time-lapse experiments were performed with the two complemented strains (named OatA^−/^OatA^WT^ and OatA^−/^OatA^D510A/S511A^) in the absence of nisin in order to evaluate their ability to restore the uncoupling between elongation and septation. Surprisingly, both constructs were able to revert the cell cycle defect by restoring the elongation arrest in the *oatA* mutant with a maximal length increase during division which is not significantly different than the wild type (Figure 2B, A ^−/^A^+^ and A^−/^A*). These results suggest that the absence of the OatA protein itself, but not a deprivation of MurNAc *O*-acetylation is responsible for the observed phenotype.

### The Transmembrane Domain of OatA Localizes at a Septal Position and is Sufficient to Rescue the Cell Cycle Defect of the OatA-deficient Strain

**Figure 3:**
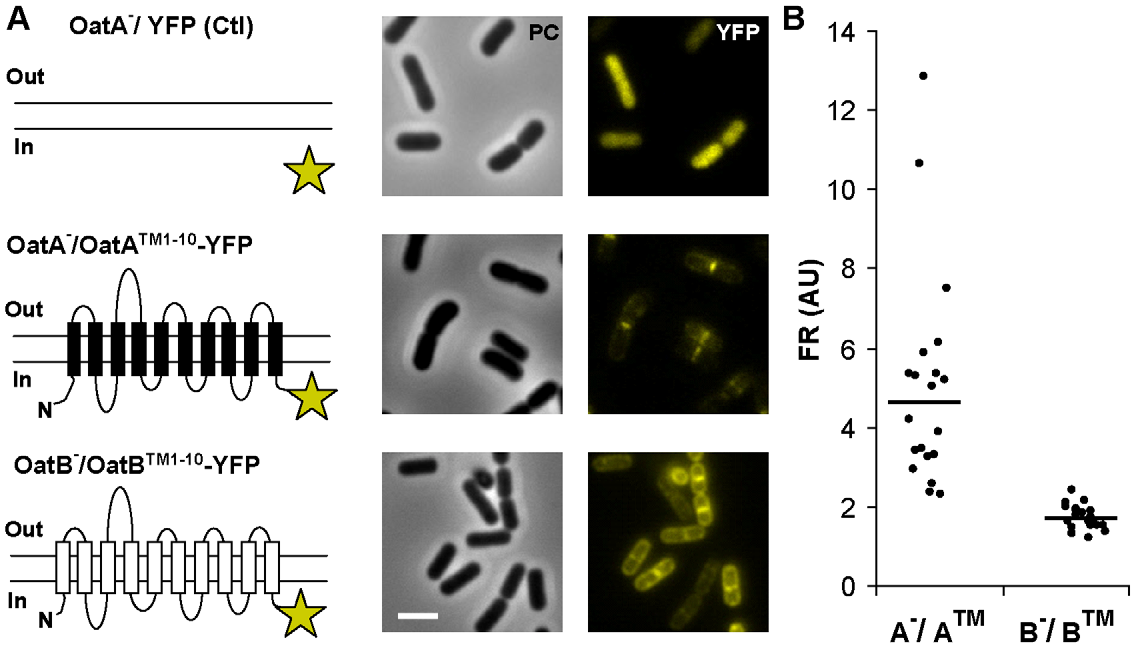
Subcellular localization of OatA^TM10^-YFP and OatB^TM10^-YFP fusions. A) Schematic representation of fusion proteins (YFP, yellow star) and corresponding micrographs in phase contrast (PC) microscopy and fluorescence microscopy (YFP). Upper panels, *oatA* mutant producing cytoplasmic YFP (OatA^−/^YFP) used as control; middle panels, *oatA* mutant producing OatA^TM1–10^-YFP (OatA^−/^OatA^TM1–10^-YFP); lower panels, *oatB* mutant producing OatB^TM1–10^-YFP (OatB^−/^OatB^TM1–10^-YFP). Induction of expression was performed with 10 ng/ml of nisin. Bar scale, 2.0 µm. B) Fluorescence ratio (FR; AU, arbitrary unit) between the fluorescence measured at mid-cell position and pole. A^−/^A™, *oatA* mutant producing OatA^TM1–10^-YFP and B^−/^B™, *oatB* mutant producing OatB^TM1–10^-YFP. Lines represent the mean value (n = 20, 3 independent replicates).

In order to investigate OatA subcellular localization and its possible connection to the cell cycle, a YFP (Venus) fusion protein was constructed. To ensure a functional fluorescent fusion, the first 10 transmembrane (TM) segments of OatA were fused to the N-terminus of YFP (OatA^TM1–10^-YFP) in order to keep the fluorescent moiety inside the cell and ensure proper folding (Figure 3A). This fusion protein lacks the last predicted C-terminal TM segment and the O-acetyltransferase catalytic domain of OatA [3]. To assess its ability at rescuing the cell-cycle defect of the *oatA* mutant, complementation and time-lapse experiments were performed with the truncated OatA^TM1–10^-YFP protein as reported above for the wild type and the OatA^D510A/S511A^ catalytic mutant. Intriguingly, the OatA^TM1–10^-YFP fusion was able to complement the cell cycle defect of the mutant, showing that the first 10 TM segments of OatA are sufficient to restore the wild-type phenotype (Figure 2B).

**Figure 4:**
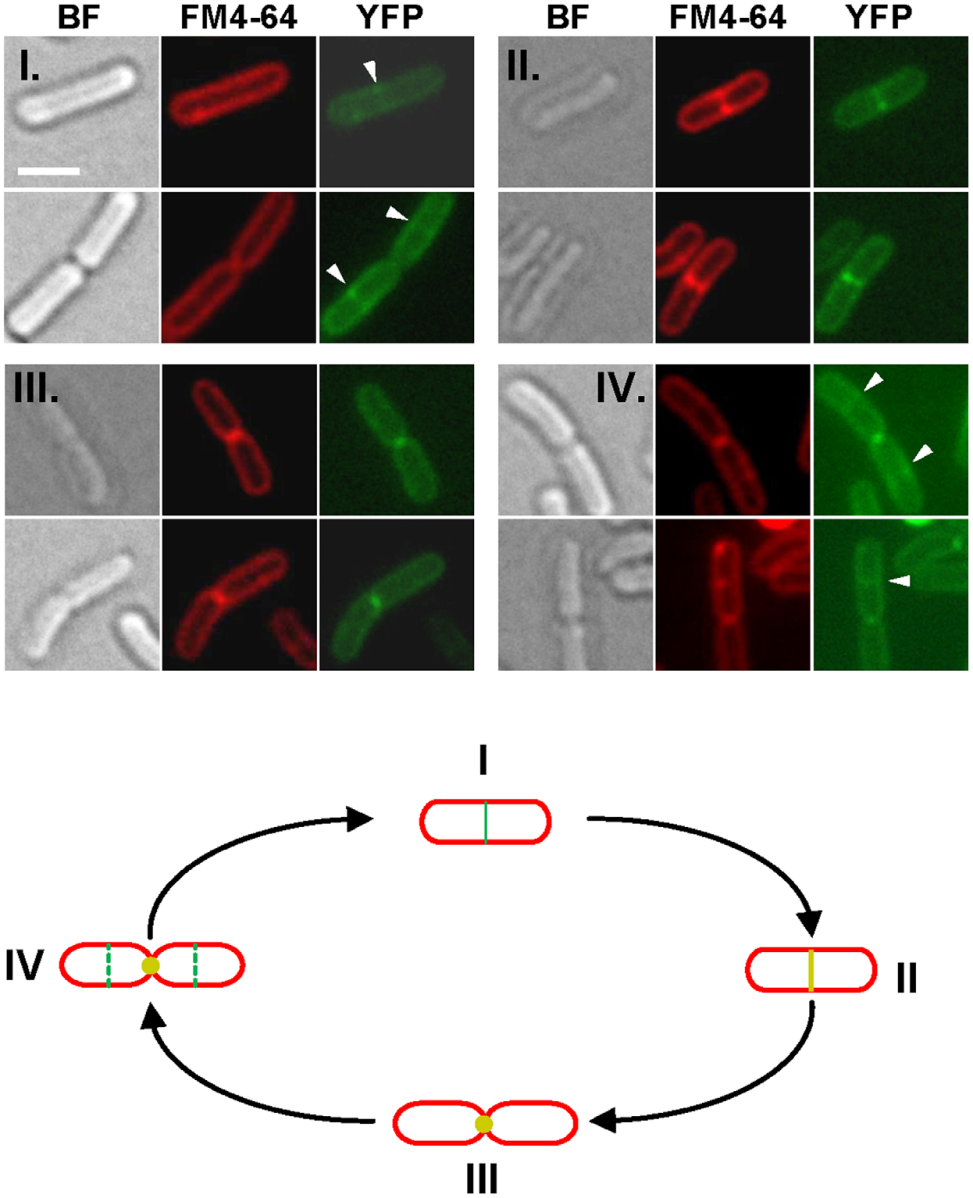
Temporal localization of OatA^TM1–10^-YFP during the cell cycle. Upper panels, selection of cells at different division stages observed in bright field (BF) microscopy and fluorescent microscopy (FM4–64, membrane staining; YFP, OatA^TM1–10^-YFP fluorescence). Induction of *oatA^TM1–10^::yfp* expression was performed with 10 ng/ml of nisin. I, septal localization of the YFP fusion prior to membrane invagination; II, co-localization of the YFP fusion and the septal membrane; III, polar localization at the end of septation; IV, reinitiation of septal localization in daughter cells. Bar scale, 1.0 µm. Lower panel, schematic representation of the cell cycle. Colors and numbers refer to above micrographs. Yellow represent a merge between FM4–64 and YFP fluorescences.

Fluorescent microscopy was then used to reveal the sublocalization of OatA^TM1–10^-YFP. A similarly constructed fusion protein between OatB and YFP (OatB^TM1–10^-YFP) as well as a cytoplasmic-localized free YFP, were used as a control. The OatB protein displays a predicted topological organization that is similar to OatA with 11 TM segments and a surface-exposed *O*-acetyltransferase domain [3]. While OatA is involved in MurNAc *O*-acetylation, OatB is responsible for GlcNAc *O*-acetylation in *L. plantarum* [3]. Fluorescent microscopy revealed different localization patterns for these fluorescent proteins. OatB^TM1–10^-YFP harbored a global membrane-associated localization pattern (OatB^−/^OatB^TM1–10^-YFP) compared to the cytoplasmic-localized YFP, whereas OatA^TM1–10^-YFP was even more restricted to a septal location (OatA^−/^OatA^TM1–10^-YFP) (Figure 3A). Fluorescence ratios (FR) between the septum and the pole were determined (Figure 3B). In the case of the OatB fusion, the measured mean fluorescence ratio was ∼2 as expected for a global membrane localization, while the mean ratio was largely higher than 2 for the OatA fusion, showing that the increased fluorescence observed at the septum was not due to the presence of the septal double membrane, but well to a specific location of OatA^TM1–10^-YFP. Due to low fluorescence level and quick bleaching of the YFP fusion, we were unable to perform time lapse experiments with the OatA/B^TM1–10^-YFP fusions. However, the localization of the OatA fusion was followed during the cell cycle by selecting of a set of cell images at different stages of growth (Figure 4). The OatA fusion could be detected at mid-cell before the invagination of the septal membrane as revealed by FM4–64 fluorescent labeling (Figure 4, stage I). After membrane invagination and septum formation, OatA^TM1–10^-YFP fluorescence formed a marked transversal band (Figure 4, stage II) that shrunk as constriction proceeded. At a late septation stage, cells retained a polar localization of the OatA fusion (Figure 4, stage III) that persisted until the next division cycle of daughter cells under fast growth conditions (Figure 4, stage IV). These data suggest that OatA is recruited at a septal position in the early stage of the division process and that it remains at the septum until the ultimate stage of division before cell separation.

### Overexpression of *oatA* Leads to Curved Cells and Aberrant Septation

As OatA is localized at a septal position during all steps of the division process and as its depletion affects the uncoupling of the elongation-septation process, we next investigated the impact of its overproduction on cell morphology. The three versions of the *oatA* gene (*oatA* ^WT^, *oatA* ^D510A/S511A^, and *oatA* ^TM1–10^ *::yfp*) were overexpressed by a high concentration of the nisin inducer in wild type and *oatA* mutant strains. The empty vector and the *oatB* ^TM1–10^ *::yfp* fusion were used as a control.

**Figure 5:**
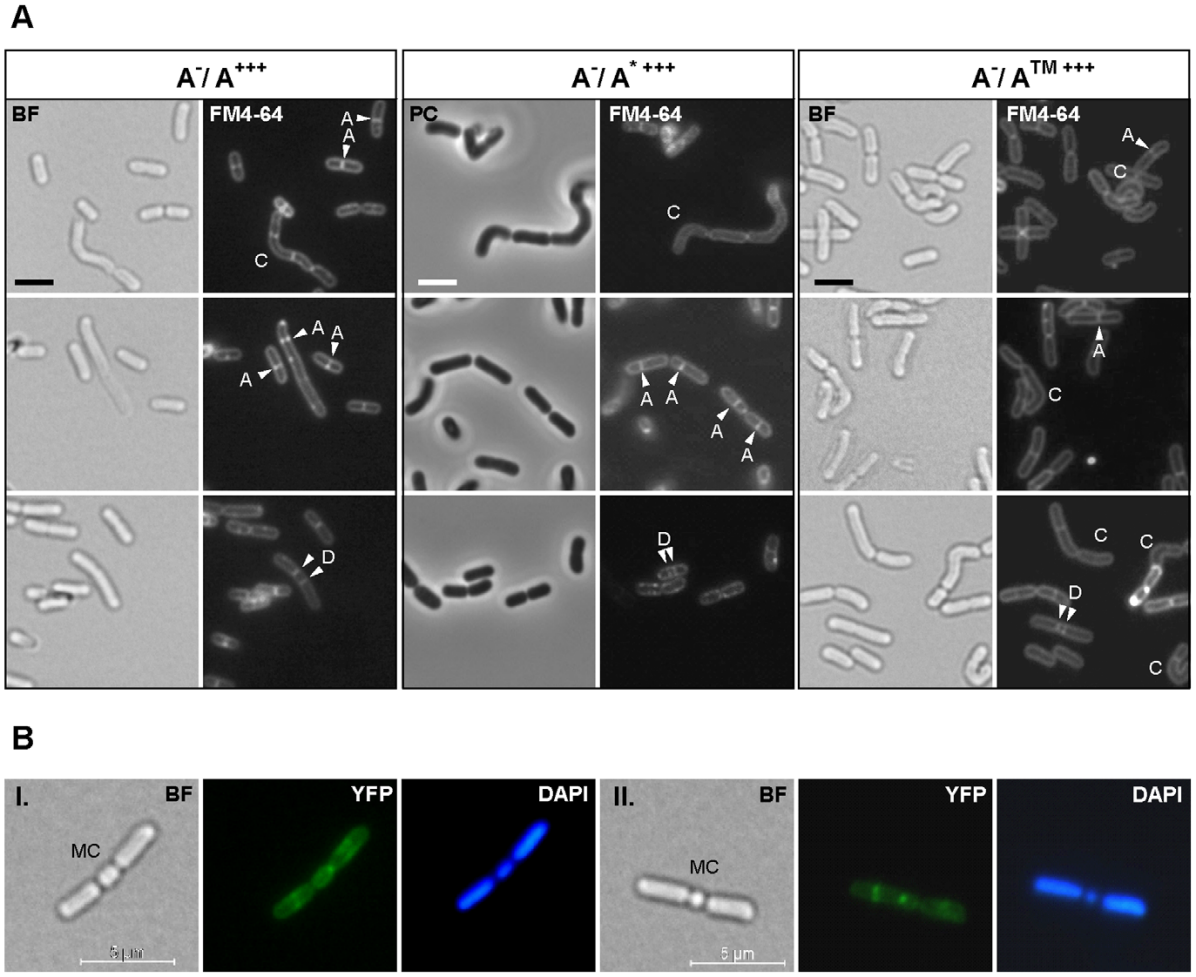
Morphological aberrations induced by the overproduction of different variants of OatA in the *oatA* mutant. Induction was performed with 20 ng/ml of nisin. A) Selection of cells showing curvature (labeled C), asymmetrical septation (labeled A), dual septation (labeled D) observed in bright field (BF) or phase contrast (PC) microscopy and fluorescent microscopy (FM4–64, membrane staining). A^−/^A^+++^, *oatA* mutant overexpressing *oatA* ^WT^; A^−/^A^* +++^, *oatA* mutant overexpressing *oatA* ^D510A/S511A^; A^−/^A™^ +++^, *oatA* mutant overexpressing *oatA* ^TM1–10^ *::yfp*. Bar scale, 2.0 µm. B) Selection of two tripartite cells (I and II) showing a central mini-cell (labeled MC) resulting from a dual septation event observed in bright field (BF) microscopy and fluorescent microscopy (YFP, OatA^TM1–10^-YFP fluorescence; DAPI, DNA staining).

Surprisingly, although *oatA* deletion did not lead to any obvious cell morphology defect, except for the lack of cell length control, overexpression of the three OatA variants led to similar morphological aberrations in a significant part of the population in both wild type and OatA^−^ backgrounds (∼ 40%–60%; e.g. WT/OatA^WT^ and OatA^−/^OatA^WT^, 45% (n = 176, 4 replicates) and 50% (n = 165, 3 replicates) abnormal cells, respectively) (Figure 5 and Figure S2). None of these morphological defects could be observed with the empty vector or the overexpression of the *oatB* ^TM1–10^ *::yfp* fusion (data not shown and Figure S3). The most obvious morphological aberrations were the presence of curved cells (e.g. WT/OatA^WT^ and OatA^−/^OatA^WT^, 16% and 22% of the global population, respectively) or cells harboring an asymmetrical septum (e.g. WT/OatA^WT^ and OatA^−/^OatA^WT^, 20%) (Figure 5A, arrows labeled C and A, respectively). More strikingly, cells with a double septum were also observed (e.g. WT/OatA^WT^ and OatA^−/^OatA^WT^, 9% and 7%, respectively) (Figure 5A, labeled D). As a possible consequence of this latter event was the presence of cell triads forming a “bow tie”-like arrangement with a mini-cell at mid-cell position (Figure 5B). The observation of the same cell aberrations for the overproduction of wild-type OatA, the catalytically inactive OatA^D510A/S511A^ mutant, and the OatA^TM1–10^-YFP fusion supports the conclusion that it is the transmembrane domain of the protein that contributes to the spatio-temporal control of septation in *L. plantarum* rather than the extent of MurNAc *O*-acetylation catalyzed by the enzyme.

### Overexpression or Deletion of *oatA* Does Not Affect the Global WTA Content

We have recently reported that *L. plantarum* WTAs are mainly localized in the cylindrical part of the cell and that they are not detectable at the septum of dividing cells [2]. This subcellular distribution of WTA is thus opposed and complementary to that reported here for OatA. In addition, a *tagO*-deleted mutant of *L. plantarum*, completely depleted in WTA, and the overproducing-OatA mutant share common morphological aberrations (i.e. curved cells and asymmetrical septation) (Figure S4, [2]). Since WTA and *O*-acetylation are both covalently linked to the C6-OH of MurNAc, we tested the impact of OatA overproduction or its depletion on WTA content. As phosphorus is abundant in WTA, the phosphorus content of the cell wall was assessed by X-ray photoelectron spectroscopy (XPS), a technique that analyses the chemical composition of the outermost cell surface [2]. Deletion or over-expression of *oatA* had no impact on the chemical composition of the external cell-wall layer of *L. plantarum* (Table 2), indicating that the global content of WTA at the cell surface was not altered by the modulation of MurNAc *O*-acetylation. Additionally, the analysis of the cell surface of the different mutants by atomic force microscopy (AFM) showed that the deletion or over-expression of *oatA* had no impact on the cell nanomorphology (Figure S5). Both mutants maintained a highly polarized cell surface with a rough cylindrical part and smoother poles as was found for wild type *L. plantarum*. In the case of the *tagO* mutant, we previously reported that the cell surface is more homogenous between the poles and the cylindrical part (loss of polarization) resulting from the absence of WTA in the cylindrical part (Figure S5, [2]). Altogether, these results suggest that the content of WTAs and their cell wall distribution are not significantly affected by the level of MurNAc *O*-acetylation.

**Table 2:** Surface chemical composition of *L. plantarum* strains measured by XPS, and proportions of carbon involved in peptides (C_Pe_), glycans (C_Gl_) and lipids (C_Lp_) deduced from the data.

strain	%C	%O	%N	%P	%C_Pe_	%C_Gl_	%C_Lp_
WT	60.8	35.5	2.9	0.8	17	63.5	19.4
WT/OatA^WT^	60.5	34.4	4.1	0.9	24.6	58.7	16.6
OatA^−^	56.1	37.8	2.2	0.6	14.4	75.3	10.2
OatA^−^/OatA^WT^	59.0	37.0	3.1	0.8	19.1	68.0	12.9
OatA^−^/OatA^D510AS511A^	59.1	37.4	2.9	0.8	17.8	69.1	13.0
TagO^−^ #	60.5	36.3	2.7	0.1	16.3	65.8	17.8

# data from André et al. [2].

### The Inhibition of Division by the Min System is Disturbed in the *oatA* Mutant

**Figure 6:**
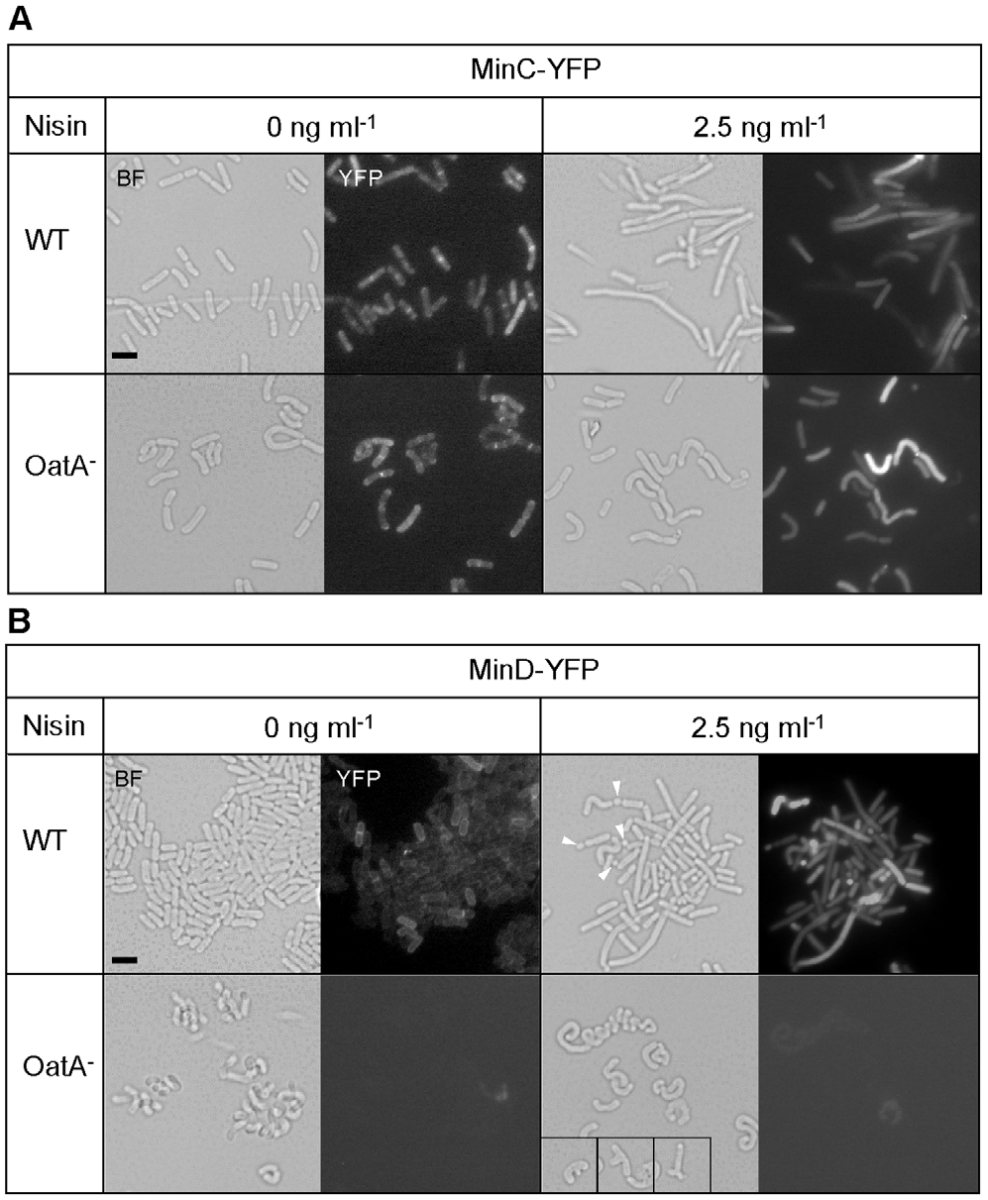
Effect of expression of *min::yfp* fusions on cell morphology and subcellular localization. Effect of expression of *minC::yfp* (A, MinC-YFP) and *minD::yfp* (B, MinD-YFP) in *L. plantarum* wild-type (WT) and *oatA* mutant (OatA^−^) without nisin induction (0 ng/ml) and with 2.5 ng/ml of nisin. Micrographs were obtained in bright field (BF) microscopy and fluorescence microscopy (YFP). For MinD-YFP (nisin 2.5 ng/ml), minicells are indicated by arrows in WT and three selected branched cells (insets) are added for OatA^−^. Bar scale, 2.0 µm.

In *B. subtilis*, variation in the level of MinCD division inhibitors was shown to result in misplaced septa with mini-cell production at the poles, cell elongation, and a loss of cell cycle synchrony [23], [24]. In order to determine whether the Min system is disturbed in the *oatA* mutant, we examined MinC and MinD localization by using YFP fusions and compared the impact of their overproduction in wild-type and *oatA* mutant cells of *L. plantarum* (Figure 6).

In wild-type cells, MinC-YFP and MinD-YFP fusions localized at the poles and at mid-cell under low expression conditions (i.e. in the absence of nisin) and showed a cytoplasmic distribution upon overproduction by nisin induction (2.5 ng/ml). Overproduction of either MinC-YFP or MinD-YFP in the wild-type resulted in filamentation as expected from inhibition of cell division (Figure 6). In addition, mini-cells (Figure 6B, arrows) and asymmetrical septa were observed for MinD-YFP. Globally, these observations are consistent with previous data obtained in *B. subtilis* concerning MinCD sublocalization and cell elongation by MinCD overproduction [24], [25].

In the *oatA* mutant, the MinC-YFP shows a similar localization as observed in the wild-type while fluorescence of the MinD-YFP fusion is hardly detected at both nisin concentrations (0 and 2.5 ng/ml) (Figure 6B) and could only be observed at a much higher level of induction (10 ng/ml) (data not shown). This could indicate that the targeting or stability of MinD-YFP is affected in the *oatA* mutant. Remarkably, overproduction of both fusions in the *oatA* mutant led to curved/twisted cells as well as to a range of branched or spiral-shaped cells in the case of MinD-YFP, which strongly contrast with the filamentation phenotype observed in the wild type (Figure 6B). Moreover, curved cells are already observed in absence of nisin while the cell morphology of the wild type is globally unaffected.

These observations suggest that the Min system acts differently in the absence of OatA by altering the activity or the position of the division machinery resulting in a range of cell shape abnormalities.

## Discussion

We have previously reported that the MurNac *O*-acetylation performed by OatA in *L. plantarum* plays an important role in the resistance of peptidoglycan to hydrolysis [3]. Here, we show that the OatA protein plays a key role in the spatio-temporal control of the cell cycle in *L. plantarum*. The absence of OatA leads to a loss of the uncoupling between elongation and septation, whereas its overproduction results in mislocated septa. Importantly, we show that the OatA protein itself, but not a depletion of MurNAc *O*-acetylation is responsible for the observed phenotypes.

It is well established that cell growth of species such as *E. coli* or *B. subtilis* is discontinuous by the periodic succession of elongation and septation events [26]–[28]. In order to maintain a mean cell size in the bacterial population, a “sensing mechanism” must be present to ensure that cell division takes place when the cell has doubled in length. However, this mechanism is poorly understood except for the importance of the metabolic effector UgtP that transmit the nutrient status of the cell to the division machinery in *B. subtilis*, so coupling nutrient availability and cell length during growth [26], [27]. The continuation of elongation during the septation process and the resulting length heterogeneity of OatA-deficient cells of *L. plantarum* suggest that this ‘sensing mechanism’ is affected. The presence of OatA at mid-cell in the early stage of the division process and the capacity of OatA to delocalize the septum formation when overproduced indicate that OatA is somehow involved in the spatio-temporal recruitment of the divisome at mid-cell.

One of the mechanisms contributing to the timing of cell division that could be altered in the *oatA* mutant is the MinCDJ/DivIVA system. One intriguing observation was the ability of OatA and its mutated variants to directly or indirectly be responsible of the formation of a second septum through their overproduction. The two septa are localized at nearly mid-cell, leading to the formation of a central mini-cell. The production of mini-cells is reminiscent of the *minCD* mutant phenotype of *B. subtilis* but in that case, anucleated mini-cells were localized at the poles. In *B. subtilis*, the role of MinCD is to disassemble the old divisome at the new poles and to prevent assembly of Z rings at poles [23]. The recruitment of the division inhibitor MinCD at the pole is determined by MinJ, which bridges DivIVA and MinD [29]. It was recently shown that DivIVA localization depends on membrane curvature and is recruited at a septal localization since the curvature of the membrane invagination during the septation is higher than any elsewhere in the cell [30]. As OatA appears to be recruited at a mid-cell position in the early stage of the division process, we cannot exclude an effect of OatA on DivIVA localization with an impact on the functioning of the Min system. Unfortunately, we were unable to obtain a functional DivIVA-YFP fusion to assess the impact of OatA on DivIVA localization. Nevertheless, the modulation of the stoichiometry between MinC and MinD was achieved by the overproduction of MinC- or MinD-YFP fusions. While the overproduction of MinC- or MinD-YFP fusions led to cell elongation in the wild type strain as expected, their overproduction in the *oatA* mutant had a more severe effect giving a high proportion of curved cells and aberrant cell morphologies. All these cell aberrant morphologies, such as Y-, C- or T-shaped cells and corkscrew filaments, are reminiscent of morphological anomalies observed in *fts* mutants of various bacterial species where an alteration of the activity or position of FtsZ has been proposed as a common theme [31]. Our observations based on MinC/MinD overproduction are an additional indication that the activity or position of the divisome is altered in the *oatA* mutant.

Interestingly, OatA and WTA harbor opposite and reciprocal sub-cellular distributions in *L. plantarum*. While WTA were shown to be absent from the septum, at least in their mature form [2], we show here that OatA localizes at a septal position. In addition, complete depletion of WTA was reported as leading to misplaced septa, suggesting that WTA are involved in the correct positioning of the division machinery. Here, we report that OatA overproduction leads to similar cell aberrations. Altogether, these observations suggest that the septum could be an *O*-acetylated MurNAc-rich and WTA-poor region. As MurNAc *O*-acetylation and WTA are competitive substituents of MurNAc, their anchoring to the PG has to be regulated and their relative ratio could be of high importance for proper morphogenesis. However, surface analysis showed that the global WTA content is not affected by the amount of *O*-acetylated MurNAc. Nevertheless, it is striking that both OatA overproduction and TagO inactivation results in similar cell morphotypes. An excess of OatA at the septum could alter either the temporal synthesis or the abundance of immature septal WTAs that could be important morphogenesis determinants since recently shown to directly recruit a penicillin-binding protein at a septal position [16]. In addition to the Min system, a mechanism sensing the presence of WTA (or its synthesis machinery) and OatA for the spatio-temporal control of the division process could be hypothesized.

To conclude, we have shown the involvement of the OatA protein in three septation-related mechanisms in *L. plantarum*: i. OatA is needed for the uncoupling between elongation and septation phases, ii. OatA has the capacity to interfere with septum position and to induce the formation of aberrant septa, and iii. OatA is required for the appropriate inhibition of the division process by the Min system. Altogether, OatA seems to be an important actor of readiness to divide. Future work will be dedicated to identify potential partners of OatA, especially proteins involved in the early steps of divisome assembly, and to follow their temporal localization by time-lapse experiments during the cell cycle.

## Supporting Information

Figure S1 **Study of the synchrony between elongation and septation phases by time-lapse experiments.** Cell length (µm) of the mother cell during the division process for the wild type (WT) and the *oatA* mutant (OatA^−^) is indicated on the micrographs. Time (min) was arbitrarily fixed to zero at the last view before any detectable cell invagination in bright field.
(PDF)

Figure S2 **Morphological aberrations induced by the overproduction of OatA and OatA^TM1–10^-YFP in the wild-type strain.** Induction was performed with 20 ng/ml of nisin. Selection of cells showing curvature (labeled C), asymmetrical septation (labeled A), dual septation (labeled D) observed in phase contrast (PC) microscopy and fluorescent microscopy (FM4–64, membrane staining; DAPI, DNA staining). WT/A^+++^, wild type overexpressing *oatA* ^WT^; WT/A™^ +++^, wild type overexpressing *oatA* ^TM1–10^ *::yfp*.
(PDF)

Figure S3 **Comparison of the overproduction of OatA^TM1–10^-YFP and OatB^TM1–10^-YFP.** Induction was performed with 20 ng/ml of nisin. A^−/^A™^ +++^, *oatA* mutant overexpressing *oatA* ^TM1–10^ *::yfp*; B^−/^B™^ +++^, *oatB* mutant overexpressing *oatB* ^TM1–10^ *::yfp*; WT/B™^ +++^, WT *oatB* mutant overexpressing *oatB* ^TM1–10^ *::yfp*, and WT/pNZ8048, WT carrying the empty plasmid. Asterisks indicate a range of aberrant morphologies in A^−/^A™^ +++^. Bar scale, 2.0 µm.
(PDF)

Figure S4 **Morphological aberrations observed in the** ***tagO*** **mutant.** Selection of cells showing curvature (labeled C) and asymmetrical septation (labeled A) observed in fluorescent microscopy (FM4–64, membrane staining). Bar scale, 2.0 µm
(PDF)

Figure S5 **AFM topographic images of WT and mutant cells.** Height images (a, d, g, j), deflection images (b, e, h, k) and vertical cross-sections (c, f, i, l) taken along the dotted line shown on the height images recorded in sodium acetate buffer for *L. plantarum* WT cells (a, b, c), OatA^−^ mutant cells (d, e, f), OatA^−/^OatA^D510AS511A^ mutant cells (g, h,i), and for TagO^−^ mutant cells (j, k, l).
(PDF)

Table S1 Primers used for cloning and validation.
(PDF)
