# Supplementary figures and images for: Dual Role for the O-Acetyltransferase OatA in Peptidoglycan Modification and Control of Cell Septation in Lactobacillus plantarum
Source: PLoS One. 2012 Oct 26;7(10):e47893. doi: 10.1371/journal.pone.0047893 (PMC3482227; doi:10.1371/journal.pone.0047893)

**Figure S1.**

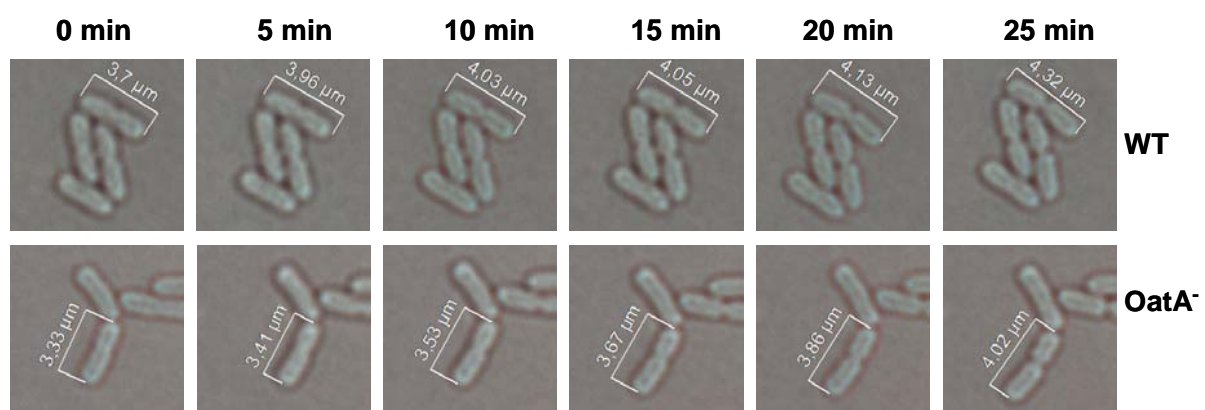

Supplement: Figure S1 — Study of the synchrony between elongation and septation phases by time-lapse experiments. Cell length (µm) of the mother cell during the division process for the wild type (WT) and the oatA mutant (OatA−) is indicated on the micrographs. Time (min) was arbitrarily fixed to zero at the last view before any detectable cell invagination in bright field. (PDF) [file pone.0047893.s001.pdf]

Figure S2.

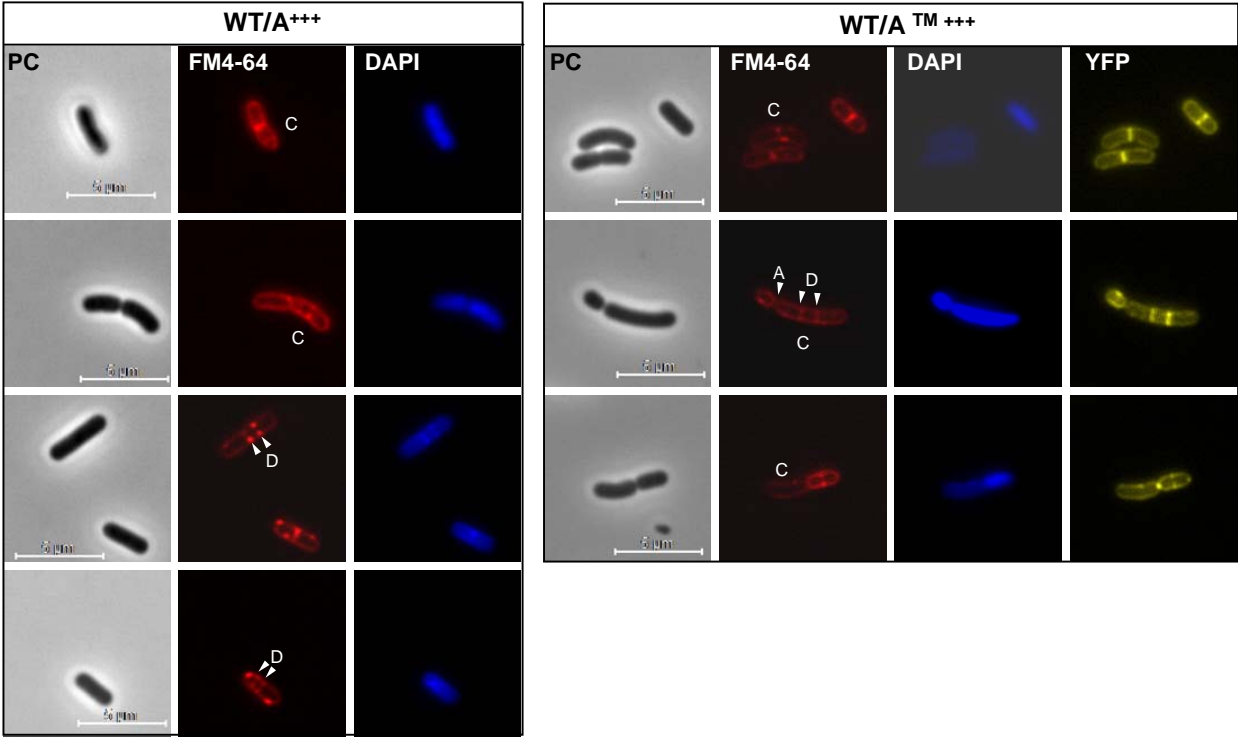

Supplement: Figure S2 — Morphological aberrations induced by the overproduction of OatA and OatATM1–10-YFP in the wild-type strain. Induction was performed with 20 ng/ml of nisin. Selection of cells showing curvature (labeled C), asymmetrical septation (labeled A), dual septation (labeled D) observed in phase contrast (PC) microscopy and fluorescent microscopy (FM4–64, membrane staining; DAPI, DNA staining). WT/A+++, wild type overexpressing oatA WT; WT/A™ +++, wild type overexpressing oatA TM1–10 ::yfp. (PDF) [file pone.0047893.s002.pdf]

Figure S3.

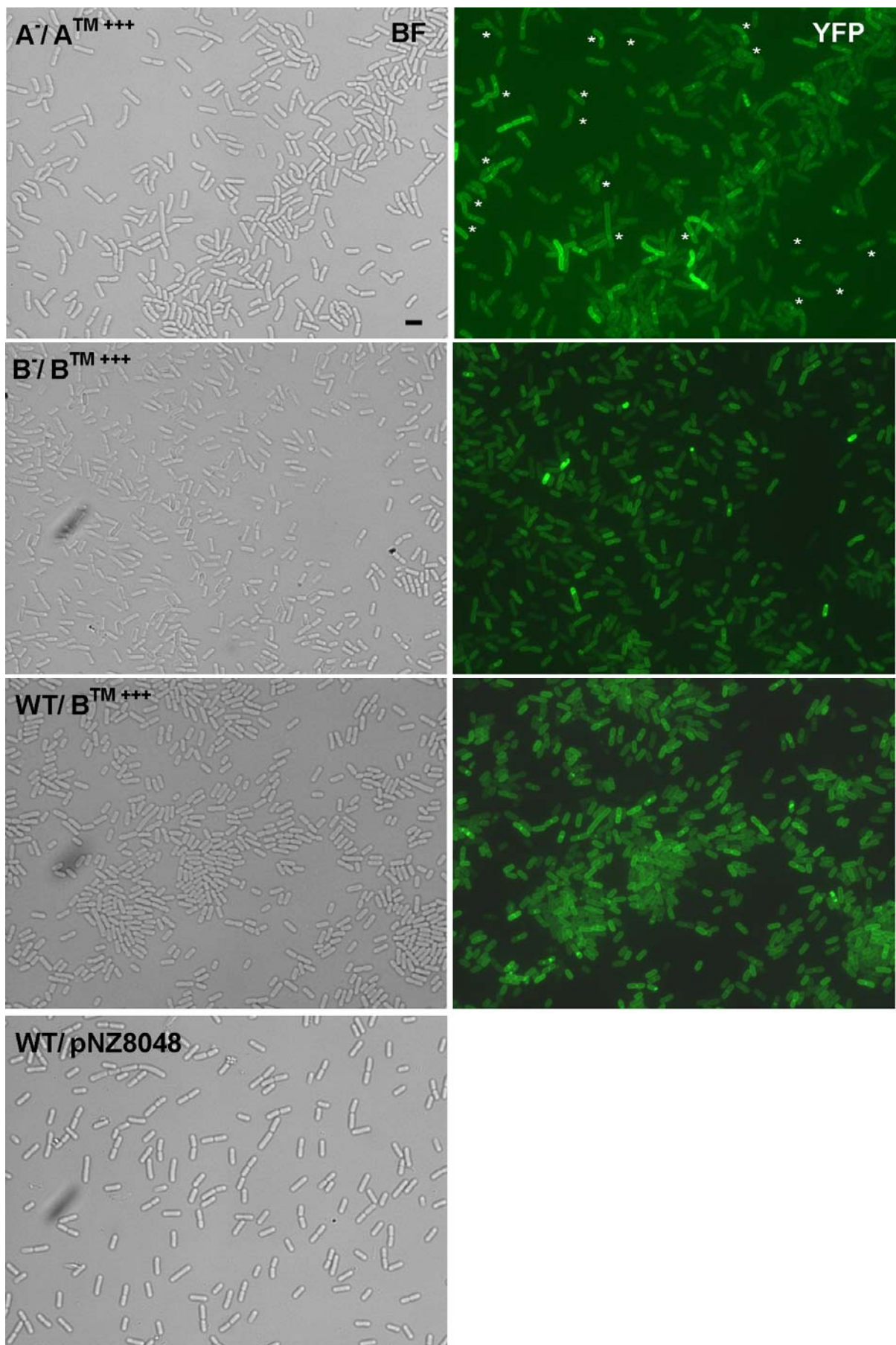

Supplement: Figure S3 — Comparison of the overproduction of OatATM1–10-YFP and OatBTM1–10-YFP. Induction was performed with 20 ng/ml of nisin. A−/A™ +++, oatA mutant overexpressing oatA TM1–10 ::yfp; B−/B™ +++, oatB mutant overexpressing oatB TM1–10 ::yfp; WT/B™ +++, WT oatB mutant overexpressing oatB TM1–10 ::yfp, and WT/pNZ8048, WT carrying the empty plasmid. Asterisks indicate a range of aberrant morphologies in A−/A™ +++. Bar scale, 2.0 µm. (PDF) [file pone.0047893.s003.pdf]

**Figure S4**

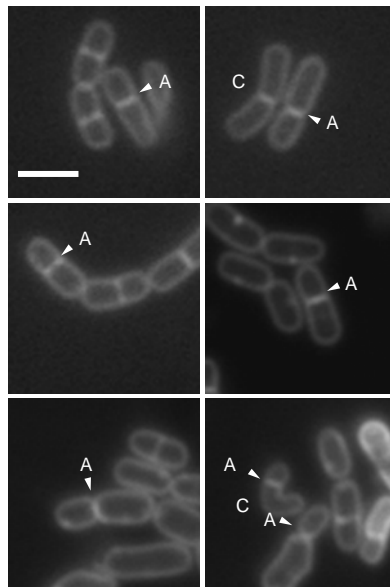

Supplement: Figure S4 — Morphological aberrations observed in the tagO mutant. Selection of cells showing curvature (labeled C) and asymmetrical septation (labeled A) observed in fluorescent microscopy (FM4–64, membrane staining). Bar scale, 2.0 µm (PDF) [file pone.0047893.s004.pdf]

**Figure S5.**

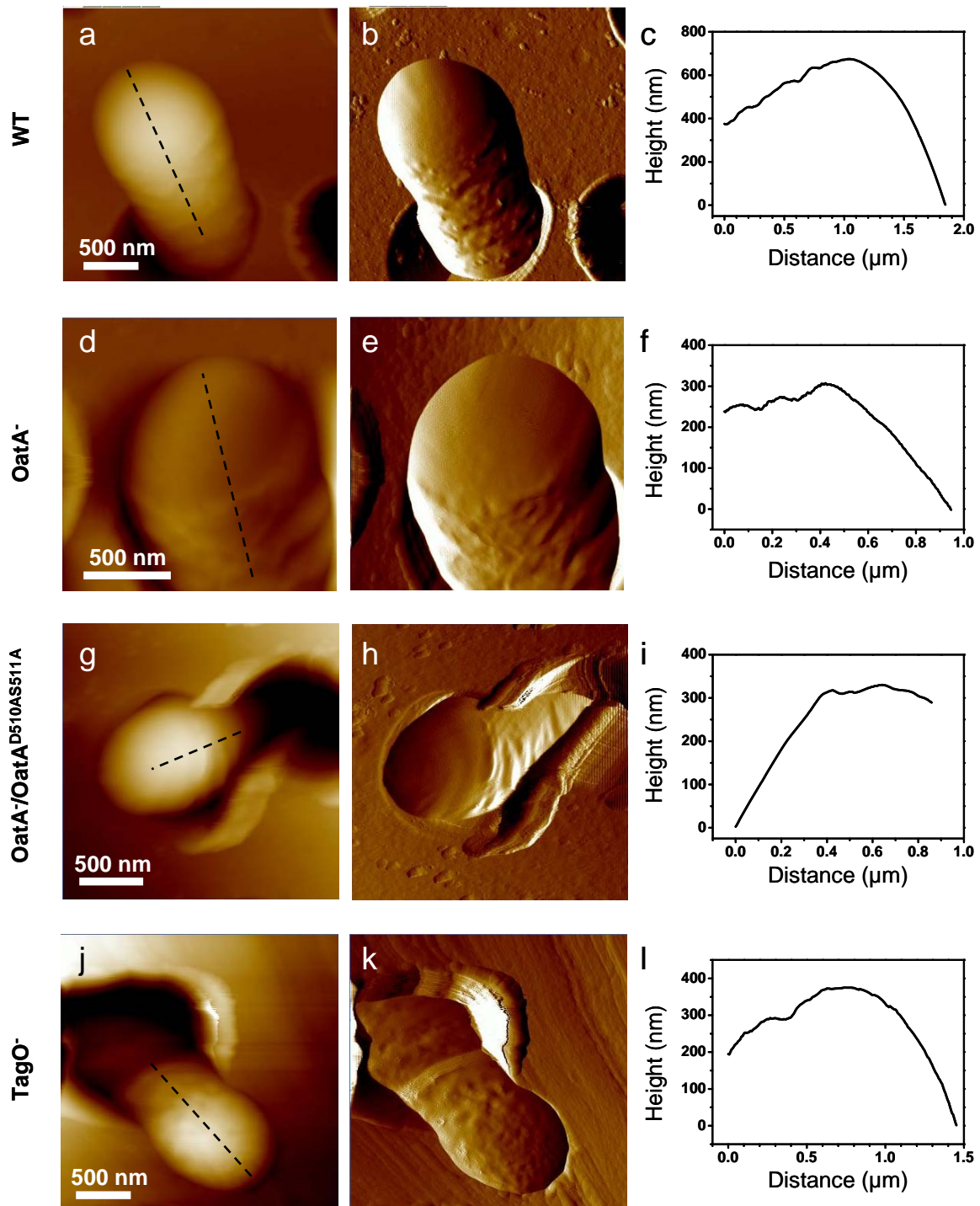

Supplement: Figure S5 — AFM topographic images of WT and mutant cells. Height images (a, d, g, j), deflection images (b, e, h, k) and vertical cross-sections (c, f, i, l) taken along the dotted line shown on the height images recorded in sodium acetate buffer for L. plantarum WT cells (a, b, c), OatA− mutant cells (d, e, f), OatA−/OatAD510AS511A mutant cells (g, h,i), and for TagO− mutant cells (j, k, l). (PDF) [file pone.0047893.s005.pdf]
