# Supplementary material for: Dual Role for the O-Acetyltransferase OatA in Peptidoglycan Modification and Control of Cell Septation in Lactobacillus plantarum
Source: PLoS One. 2012 Oct 26;7(10):e47893. doi: 10.1371/journal.pone.0047893 (PMC3482227; doi:10.1371/journal.pone.0047893)
Supplement: Table S1 — Primers used for cloning and validation. (PDF) [file pone.0047893.s006.pdf]

Table S1. Primers used for cloning and validation

Primer name	Sequence[a]	Mutant strain or plasmid
**Primers used for the construction of the YFP reporter vector**		
NcoIVenus4X4	5'-AACCATGGACGTCTCTAGACCTGCAGGCGCATGCGTGAGCAAGG GCGAGGAGCTGTTCACCGGGGT-G 3'	pGIBD008
SacIVenus4X4	5' –AAAGAGCTCTTAGGATCCGGAACTAGTAACGCCCGGGCACATG TGGCGCGCAGCACGCGTCTTGTACAGCTCGTCCATGC-3'	
**Primers used for the construction of YFP fusions**		
5'OatAPstI	5’-ATACTGCAGGAAAATTTGCGGAGCG-3’	pGIEB018
3'TMLoatAXbaI	5’-TCGATCTAGACAATAACCGACTGTAAT-3’	
5'OatBPstI	5’-GTCCTGCAGCCAAAAAAATTAGGGGGACGGTAAGTTAG-3’	pGIEB019
3'TMLoatBXbaI	5’-TCGATCTAGAGCCCGCAATCACTGGTTC-3’	
5'minC_NcoI	5’-ATGCCATGGAACAGAGTGTAGTTTTAAAG-3’	pGIEB020
3'minC_XbaI	5’-TCGATCTAGAAACACGTCCTCCATTTCG-3’	
5'minD_NcoI	5’-ATGCCATGGGAAAAGCAATTGTCATCAC-3’	pGIEB021
3'minD_XbaI	5’-TCGATCTAGACTTGCCACGATGAAAAATTC-3’	
**Primers used for the validation of the constructed vectors**		
pNisA1	5’-TACTGACAATAGAAACATTAAC-3’	pNZ8048 and derivatives
pNisA2	5’-TATCAATCAAAGCAACAC-3’	

[a] Restriction sites introduced in the primers are underlined
